# Barriers to accessing and using health insurance cards among methadone maintenance treatment patients in northern Vietnam

**DOI:** 10.1186/s13011-017-0119-0

**Published:** 2017-07-17

**Authors:** Bach Xuan Tran, Victoria L. Boggiano, Cuong Tat Nguyen, Long Hoang Nguyen, Anh Tuan Le Nguyen, Carl A. Latkin

**Affiliations:** 1 Institute for Preventive Medicine and Public Health, Hanoi Medical University, Hanoi, Vietnam; 2 Johns Hopkins Bloomberg School of Public Health, Baltimore, Maryland USA; 3 University of California, Berkeley School of Public Health, Berkeley, California USA; 4 Institute for Global Health Innovations, Duy Tan University, Da Nang, Vietnam; 5 School of Medicine and Pharmacy, Vietnam National University, Hanoi, Vietnam

**Keywords:** Methadone maintenance treatment, Health insurance, Barriers

## Abstract

**Background:**

Methadone maintenance treatment (MMT) patients face unique costs associated with their healthcare expenditures. As such, it is important that these patients have access to health insurance (HI) to help them pay for both routine and unforeseen health services. In this study, we explored factors related to health insurance enrollment and utilization among MMT patients, to move Vietnam closer to universal coverage among this patient population.

**Methods:**

A cross-sectional study was conducted with 1003 patients enrolled in MMT in five clinics in Hanoi and Nam Dinh provinces. Patients were asked a range of questions about their health, health expenditures, and health insurance access and utilization. We used multivariate logistic regressions to determine factors associated with health insurance access among participants.

**Results:**

The majority of participants (nearly 80%) were not currently enrolled in health insurance at the time of the study. Participants from rural regions were significantly more likely than urban participants to report difficulty using HI. Family members of participants from rural regions were more likely to have overall poor service quality through health insurance compared with family members of participants from urban regions. Overall, 37% of participants endorsed a lack of information about HI, nearly 22% of participants reported difficulty accessing HI, 22% reported difficulty using HI, and more than 20% stated they had trouble paying for HI. Older, more highly educated, and employed participants were more likely to have an easier time accessing HI than their younger, less well educated, and unemployed counterparts. HIV-positive participants were more likely to have sufficient information about health insurance options.

**Conclusions:**

Our study highlights the dearth of health insurance utilization among MMT patients in northern Vietnam. It also sheds light on factors associated with increased access to and utilization of health insurance among this underserved population. These results can help improve health insurance enrollment among MMT patients, a population that is at increased need of financial assistance in accessing health services.

## Background

Injection drug use is considered to be a primary cause of HIV transmission in most countries worldwide [1]. Methadone maintenance treatment (MMT) is regarded as a highly effective medication to treat opioid dependence [2] and is viewed as an important component of HIV/AIDS prevention strategies [3]. Methadone reduces the frequency of illicit drug use, improves treatment outcomes among HIV-positive and negative individuals, and eventually, helps to alleviate the economic burden caused by substance abuse [4].

Addiction to opiates has been well documented to constitute a chronic medical illness [5], and as such, individuals who are dependent on opiates need access to long-term medical care. People who inject drugs are at increased risk of acquiring many diseases in addition to HIV/AIDS, which require medical care and may lead to catastrophic healthcare expenditures and impoverishment [6]. This vulnerable population needs financial support to help them pay for their health coverage, the primary source of which is health insurance. Appropriate and consistent access to health insurance (HI) helps patients pay for and utilize necessary treatments such as methadone maintenance therapy (MMT) or other health care services if needed. Studies conducted in other countries have highlighted the importance of health insurance in improving opiate-addicted patients’ access to methadone treatment. One study conducted by Peterson, et al., in the United States found that opiate-addicted patients often remain out-of-treatment due to a lack of health insurance or money to be able to pay for care [7]. Importantly, MMT has been shown to lead to improved long-term outcomes for opiate-addicted patients in comparison to other treatment options [8].

Vietnam is currently pursuing the 90-90-90 HIV/AIDS target for 2020 [9] and MMT programs represent an important way for Vietnam to achieve this goal. In Vietnam between the years of 2008 and 2013, MMT treatment was provided free of charge by international donors [4]. However, in more recent years, the country has experienced reduced financial support from these sources [6, 10]. As a result, patients on MMT are now required to pay co-pays for their care as an essential resource mobilization method of the Vietnam Government [11]. However, along with the potential financial burden of methadone treatment, MMT patients in Vietnam are particularly wary of the high costs of both outpatient medical treatment and hospitalizations. Thus, health insurance has a central role in ensuring the accessibility of health service for the opiate drug users. Health insurance for MMT patients has been linked with better clinical outcomes such as lower probability of AIDS and sustained viral suppression if patients are HIV-positive, as well as overall reduced premature death [12]. Moreover, previous studies have found that drug-addicted individuals are more likely to engage in treatment if they have health insurance to help them alleviate some of the costs [7].

For the past several decades, Vietnam has been moving toward universal health insurance coverage [13, 14]. Indeed, the government has greatly expanded health insurance coverage and increased subsidies to poor and ethnic minorities across the country [15]. However, there are many barriers to achieve this goal in the country, including limited affordability, low-quality and fragmented insurance options, and no comprehensive legal system for health insurance [16]. Indeed, while health insurance in Vietnam has been shown to increase inpatient services among poor citizens, it has not been shown to greatly increase outpatient or preventive services [17]. Therefore, in the coming years, expanding health insurance coverage to vulnerable populations such as MMT patients is a priority in the Master Health Plan of the Vietnamese Government.

Previous researchers have found that level of enrollment in health insurance was associated with cost of insurance premiums, knowledge of insurance benefits, and overall affordability [18]. Other scholars have cited the importance of the country’s national and provincial governments maintaining control over health insurance funding and trying to find creative ways to increase enrollment at the provincial level [13]. In Vietnam, insured patients can access MMT freely because HI covers this service; however, patients have to own the HI card when using service. Despite national efforts to increase health insurance enrollment among the Vietnamese population, there is a dearth of studies looking at barriers to and facilitators of health insurance access among MMT patients in Vietnam. Thus, this study was conducted to better understand factors related to health insurance enrollment and utilization among MMT patients, and to propose solutions to achieve universal coverage among this patient population.

## Methods

### Study setting and population

We conducted a clinic-based cross-sectional survey from June to August 2013 in five MMT clinics in Hanoi and Nam Dinh province. To be included in the study, the clinics had to be: 1) providing methadone treatment; 2) from either an urban or rural part of northern Vietnam; and 3) covering provincial and district levels of the Vietnamese health system. We interviewed patients from Provincial AIDS Center in Nam Dinh City, District Health Center in Xuan Truong, Tu Lien, Long Bien and Regional Polyclinic in Ha Dong District.

We offered enrollment to patients who met the following criteria: 1) currently taking part in MMT treatment at one of the clinics included in the study; 2) 18 years of age and older; 3) available at clinics during the study period; and 4) able to answer the study questionnaire. After screening patients based on our selection critieria, eligible patients were invited into a private counseling room in the clinic and were informed about the purposes of the study and asked to provide their written informed consent. A convenience sampling approach was used to recruit patients. We screened 1016 patients, and all of them met the eligible criteria and agreed to participate in the study.

### Measures and instruments

We performed face-to-face interviews using structured questionnaires to collect data from the MMT patients included in the study. Interviewers were well-trained public health Masters students working in the field of substance abuse at the Hanoi Medical University. We did not involve health staff in the study, in order to avoid social desirability bias.

To address our study objectives, we asked patients to report whether they had HI and if so what types of HI; the number of people in their families with HI; and barriers to obtaining and/or using HI and their preference for when they would ideally like to pay for their HI. The barriers included: 1) Lack of information about HI; 2) Feeling difficulties in accessing to health insurance selling points; 3) Feeling difficulties in using HI to pay treatment cost; and 4) Feeling financial difficulties in paying health insurance cost.

In addition, we also collected covariates such as patients’ socio-demographic status (age, gender, education, marital status, religious beliefs, employment status); health service utilization (inpatient and outpatient care) and level of payment; and illicit drug use characteristics (drug injection, drug rehabilitation, age at on set of drug use and duration of MMT). The five-level EQ-5 dimensions-5 Levels (EQ-5D-5 L) was employed to measure the health status of respondents. This instrument measured five domains including mobility, self-care, usual activities, pain/discomfort and anxiety/depression [19].

### Statistical analysis

We performed Chi-squared tests, Fisher’s exact test and Mann-Whitney test to measure variations in variables of interest between urban and rural clinics. A *p*-value of less than 0.05 was considered statistically significance. Multivariate logistic regression was employed to identify factors associated with having HI and any additional factors that related to barriers with regards to utilizing HI among MMT patients. The reduced model was constructed using a stepwise forward selection strategy, which included variables based on the log-likelihood ratio test at a *p*-value less than 0.1, and excluded variables at *p*-values greater than 0.2 [20].

## Results

Table 1 displays the socio-demographic characteristics of the participants in our study. There was a total of 1016 respondents, most of whom were male (98.7%). The majority of patients (67.7%) lived with a spouse or partner, and 53% of whom were self-employed. The majority of the patients in our sample were 30 years and older and had at least a secondary school education. Participants used a mixture of central, provincial, district, and occasionally private healthcare services. Full payment was the most prevalent way that patients payed for both inpatient and outpatient services.

**Table 1:** Demographic characteristics of respondents

Characteristic	Rural		Urban		Total		*p*-value
	n	%	n	%	n	%	
Age^a^							
18- <25	6	4	15	1.7	21	2.1	0.3^¶^
25- < 30	21	13.9	118	13.6	139	13.7	
30- <35	36	23.8	230	26.6	266	26.2	
35- <40	33	21.9	233	26.9	266	26.2	
40- <45	27	17.9	140	16.2	167	16.4	
> =45	28	18.5	129	14.9	157	15.5	
Sex^b^							
Female	0	0	13	1.5	13	1.3	0.24^†^
Male	151	100	852	98.5	1003	98.7	
Education^c^							
≤ Elementary	30	19.9	106	12.3	136	13.4	<0.01^¶^
Secondary	87	57.6	339	39.2	426	41.9	
High	28	18.5	359	41.5	387	38.1	
> Vocational	6	4	61	7.1	67	6.6	
Marry status^d^							
Single	29	19.2	222	25.7	251	24.7	0.03^¶^
Lives with spouse/partner	116	76.8	572	66.1	688	67.7	
Divorced/widow	6	4	71	8.2	77	7.6	
Religion^e^							
No	96	63.6	800	92.5	896	88.2	<0.01^¶^
Yes	55	36.4	65	7.5	120	11.8	
Employment^f^							
Unemployed	25	16.6	236	27.3	261	25.7	<0.01^¶^
Self-employed	67	44.4	475	54.9	542	53.4	
White collar worker	1	0.7	21	2.4	22	2.2	
Workers, Farmers	54	35.8	46	5.3	100	9.8	
Other jobs	4	2.7	87	10.1	91	9	
Inpatient service use^g^							
No	141	93.4	792	91.6	933	91.8	0.45^¶^
Yes	10	6.6	73	8.4	83	8.2	
Facility for inpatient use^h^							
Central	13	24.5	84	39.4	97	36.5	<0.01^¶^
Province	8	15.1	92	43.2	100	37.6	
Others	32	60.4	37	17.4	69	25.9	
Level of payment for inpatient services^i^							
Full payment	33	64.7	157	75.9	190	73.6	0.11^¶^
Unaffordable	12	23.5	25	12.1	37	14.3	
Partial payment	6	11.8	25	12.1	31	12	
Outpatient service use^k^							
No	83	55	641	74.1	724	71.3	<0.01^¶^
Yes	68	45	224	25.9	292	28.7	
Facility for outpatient use^l^							
Central	7	7.9	89	28.3	96	23.8	<0.01^¶^
Province	13	14.6	112	35.6	125	30.9	
District	57	64	57	18.1	114	28.2	
Private	8	9	48	15.2	56	13.9	
Other	4	4.5	9	2.9	13	3.2	
Level of payment for outpatient services^m^							
Full payment	78	96.3	277	90.8	355	92	0.35^†^
Unaffordable	2	2.5	20	6.6	22	5.7	
Partial payment	1	1.2	8	2.6	9	2.3	

^¶^ *χ* ^*2*^ *test;* ^*†*^ *Fisher’s exact test*

^a^ Chi-squared test = 6.09, df = 5; ^b^ Fisher’s exact test = 0.24, df = 1; ^c^ Chi-squared test = 35.61, df = 3; ^d^ Chi-squared test = 7.38, df = 2; ^e^Chi-squared test = 103.15, df = 1; ^f^Chi-squared test = 139.34, df = 4; ^g^Chi-squared test = 0.567, df = 1; ^h^ Chi-squared test = 41.76, df = 2; ^i^Chi-squared test = 4.43, df = 2; ^k^Chi-squared test = 22.99, df = 1; ^l^Chi-squared test = 76.45, df = 4; ^m^ Fishers’s exact test = 0.35, df = 2

Health-related behaviors, health service utilization, and overall health status of respondents in urban and rural are presented in Table 2. Nearly 70% of the patients in our study had been receiving MMT therapy for greater than one year. The average number of times that participants had been to drug rehabilitation centers was 4.8 times. Participants from rural parts of northern Vietnam were statistically significantly more likely to endorse having difficulties with mobility and problems engaging in usual daily activities, as well as reported higher rates of overall pain/discomfort and anxiety/depression, than their urban counterparts. They were less likely to report ever injecting drugs or current injection drug use than participants from urban areas. Participants from rural regions were also less likely to have been to drug rehabilitation programs, and on the whole displayed shorter MMT therapy duration compared to participants from urban clinics.

**Table 2:** Behaviors, health status and health service utilization of respondents

Characteristic	Rural		Urban		Total		*p*-value
	n	%	n	%	n	%	
EQ-5D-5 L							
Having problems with mobility^a^	20	13.3	54	6.2	74	7.3	<0.01^¶^
Having problems with self-care^b^	9	6	31	3.6	40	3.9	0.17^¶^
Having problems with usual activities^c^	17	11.3	43	5	60	5.9	<0.01^¶^
Pain/Discomfort^d^	57	37.8	123	14.2	180	17.7	<0.01^¶^
Anxiety/Depression^e^	65	43.1	145	16.8	210	20.7	<0.01^¶^
HIV status^f^							
N/A	20	13.3	38	4.4	58	5.7	<0.01^¶^
Negative	124	82.1	752	86.9	876	86.2	
Positive	7	4.6	75	8.7	82	8.1	
Ever injected drugs^g^							
No	60	39.7	210	24.3	270	26.6	<0.01^¶^
Yes	91	60.3	655	75.7	746	73.4	
Current drug injection^h^							
No	134	88.7	833	96.3	967	95.2	<0.01^¶^
Yes	17	11.3	32	3.7	49	4.8	
No. of drug rehabilitations^i^							
0	15	9.9	59	6.8	74	7.3	0.02^¶^
1-5	110	72.9	565	65.3	675	66.4	
> 5	26	17.2	241	27.9	267	26.3	
Duration of MMT (months) ^k^							
< 6	32	23.5	129	15	161	16.2	<0.01^†^
6- < 12	54	39.7	131	15.2	185	18.6	
12- < 24	49	36	306	35.5	355	35.6	
24- < 36	1	0.7	177	20.6	178	17.9	
> =36	0	0	118	13.7	118	11.8	
	Mean	SD	Mean	SD	Mean	SD	
Age at first drug use ^l^	26.6	7.4	24.2	6.6	24.5	6.7	<0.01^θ^
Years of drug use ^m^	11.2	5.3	13.6	5.9	13.3	5.9	<0.01^θ^
Years of drug injection ^n^	7.9	3.9	10.5	5	10.2	4.9	0.01^θ^
No. of drug rehabilitations ^o^	3.4	3	5.1	6.6	4.8	6.3	<0.01^θ^
Duration of MMT (months) ^p^	9.3	4.9	17.7	11.2	16.5	11	<0.01^θ^

^¶^ *χ* ^*2*^; ^†^Fishers’ exact test; ^θ^Mann-whitney test

^a^ Chi-squared test = 9.33, df = 1; ^b^ Chi-squared test = 1.92, df = 1; ^c^ Chi-squared test = 9.15, df = 1; ^d^ Chi-squared test = 48.82, df = 1; ^e^ Chi-squared test = 54.16, df = 1; ^f^Chi-squared test = 20.59, df = 2; ^g^ Chi-squared test = 15.74, df = 1; ^h^ Chi-squared test = 16.00, df = 1; ^i^ Chi-squared test = 8.35, df = 2; ^k^ Fisher’s exact test = 0.00, df = 4; ^l^ Mann-Whitney z = 3.89, df = 1009; ^m^ Mann-Whitney z = −4.91, df = 1009; ^n^ Mann-Whitney z = −4.76, df = 1009; ^o^ Mann-Whitney z = −2.46, df = 1009; ^p^Mann-Whitney z = −8.46, df = 1009

Table 3 highlights current HI coverage status and barriers faced in trying to access HI among the respondents in our study. Nearly 80% of the patients in our study were uninsured. Participants from rural regions were significantly more likely than urban participants to report difficulty using HI (Chi-squared = 16.79, df = 2, *p* < 0.01). Overall, 37% of participants endorsed a lack of information about HI, nearly 22% of participants reported difficulty accessing HI, 22% reported difficulty using HI, and more than 20% stated they had trouble paying for HI. Information was also collected about the categories of HI utilized by participants’ family members. Voluntary HI was the most common type of HI used among participants’ family members, with nearly 45% of patients stating that their loved ones used voluntary HI and 29% stating that their loved ones used compulsory HI. Families from rural regions were more likely than families from urban regions to report utilizing poor HI (chi-square = 13.57, df = 1, *p* < 0.01). The majority of participants’ family members (nearly 63%) paid for HI on a quarterly basis.

**Table 3:** Current coverage and barriers to social health insurance among respondents

Characteristic	Rural		Urban		Total		*p*-value
	n	%	n	%	n	%	
Having HI^a^							
No	129	85.4	680	78.6	809	79.6	0.06^¶^
Yes	22	14.6	185	21.4	207	20.4	
Number of people in the family with HI^b^							
0	27	18.6	188	22	215	21.5	0.42^¶^
1	35	24.1	184	21.6	219	21.9	
2	39	26.9	250	29.3	289	29	
3	27	18.6	112	13.1	139	13.9	
4	9	6.2	75	8.8	84	8.4	
> =5	8	5.5	44	5.2	52	5.2	
Types of HI among family members							
Voluntary^c^	67	44.4	389	45	456	44.9	0.89^¶^
Compulsory^d^	35	23.2	260	30.1	295	29	0.09^¶^
Poor^e^	11	7.3	17	2	28	2.8	<0.01^¶^
Children under 6^f^	15	9.9	77	8.9	92	9.1	0.68^¶^
Lack of Information about HI^g^							
Yes	55	36.4	312	37.1	367	37	0.87^¶^
No	96	63.6	529	62.9	625	63	
Difficulty accessing HI^h^							
Yes	24	16.6	190	22.8	214	21.9	0.09^¶^
No	121	83.5	643	77.2	764	78.1	
Difficulty using HI^i^							
Yes	21	15.7	193	23.2	214	22.2	<0.01^¶^
No	93	69.4	420	50.5	513	53.1	
Unknown	20	14.9	219	26.3	239	24.7	
Difficulty paying for HI^k^							
Yes	30	22.1	164	19.7	194	20.1	0.5^¶^
No	84	61.8	498	59.9	582	60.2	
Unknown	22	16.2	169	20.3	191	19.8	
Expected time to pay HI (among family members)^l^							
Monthly	12	8.2	52	6.3	64	6.5	0.08^¶^
Quarterly	77	52.7	537	64.5	614	62.8	
Every six months	16	11	63	7.6	79	8.1	
Annually	19	13	71	8.5	90	9.2	
Unknown	22	15.1	109	13.1	131	13.4	

^*¶*^ *χ* ^*2*^

^a^ Chi-squared test = 3.68, df = 1; ^b^ Chi-squared test = 4.99, df = 5; ^c^ Chi-squared test = 0.02, df = 1; ^d^ Chi-squared test = 2.95, df = 1; ^e^Chi-squared test = 13.57, df = 1; ^f^ Chi-squared test = 0.17, df = 1; ^g^ Chi-squared test = 0.03, df = 1; ^h^ Chi-squared test = 2.83, df = 1; ^i^ Chi-squared test = 16.79, df = 2; ^k^ Chi-squared test = 1.43, df = 2; ^l^ Chi-squared test = 8.32, df = 4

Factors associated with use of and barriers to social health insurance among respondents are featured in Table 4. Older patients were less likely than younger patients to report a lack of information about HI. In addition, participants who had attended vocational school were less likely than illiterate participants to report a lack of information about HI. White collar workers and farmers were more likely than unemployed participants to have HI. Rich participants were more likely than their poor counterparts to both have HI and to have less difficulty accessing HI. Participants who reported difficulty using HI or who said they had an overall lack of information about HI were less likely to have HI. Patients who had been to drug rehabilitation centers in the past were more likely to obtain HI than their counterparts who had not been to rehabilitation, but they were also more likely to experience information insufficiency and have trouble accessing HI. Individuals who were HIV positive were more likely than their HIV negative counterparts to have sufficient information about HI options.

**Table 4:** Factors associated with use of and barriers to social health insurance among respondents

Characteristic	Having health insurance		Lack of information about HI		Difficulty accessing HI		Difficulty using HI		Difficulty paying for HI	
	AOR	95%CI	AOR	95%CI	AOR	95%CI	AOR	95%CI	AOR	95%CI
Gender (Female vs Male)			1.69**	0.02; 3.37	1.91**	0.43; 3.39	1.77**	0.33; 3.21	1.96***	0.52; 3.41
Age			−0.03***	−0.05; −0.01						
Education (vs Illiterate)										
Secondary			0.29*	−0.03; 0.61	0.32*	−0.04; 0.67				
> Vocational	0.63*	−0.07; 1.32	−0.76**	−1.47; −0.05						
Employment (vs Unemployed)										
White collars	1.49**	0.12; 2.86								
Workers/Farmers	0.79**	0.16; 1.43								
Income quintile (vs Poorest)										
Poor			0.35*	−0.05; 0.75					0.54**	0.12; 0.97
Rich	0.56**	0.12; 1.01	−0.34*	−0.75; 0.06	−0.52**	−0.99; −0.04				
Having problems with mobility (Yes vs No)			0.82***	0.22; 1.43	0.79**	0.18; 1.40				
HIV status (vs Negative)										
Positive			−0.75***	−1.29; −0.20						
N/A									−1.33*	−2.78; 0.12
Catastrophic expenditure (Yes vs No)	0.56**	0.02; 1.10								
Lack of Information about HI (Yes vs No)	−0.59**	−1.05; −0.13								
Difficulty using HI (Yes vs No)	0.52**	0.02; 1.03								
No. of drug rehabilitations	0.04***	0.01; 0.07	0.04***	0.01; 0.06	0.04***	0.01; 0.06				
Duration of MMT (months)	0.02*	−0.00; 0.03								

*** *p*<0.01, ** *p* < 0.05, * *p* < 0.1

## Discussion

The study explored the barriers to accessing and utilizing HI among MMT patients in Vietnam. The vast majority of MMT patients in our study were uninsured, highlighting the high prevalence of poor insurance coverage among drug-addicted individuals in northern parts of the country. We also uncovered many of the barriers they face in finding HI information, understanding how to use it, and being able to pay for it. Not surprisingly, we found that participants who were impoverished and/or unemployed were among the least likely to obtain and utilize HI. Although individuals who had recently been to drug rehabilitation centers were more likely to obtain HI, they were also at higher risk of limited access to and information about HI, which paints a murky picture of the insurance environment for these patients. Importantly, the length of time that a patient had been enrolled in MMT was found to be positively correlated to amount of information about HI.

As mentioned, our results show a high rate of uninsured MMT patients in northern Vietnam. One potential reason for this high rate of uninsured patients lies in the economic vulnerability of Vietnamese individuals enrolled in MMT. Prior research has revealed that drug users, including those enrolled in treatment, suffer from significant financial constraints that may hamper HI subscription [21]. Indeed, MMT patients are likely to spend more money on drug and health care use, and to suffer from catastrophic health expenditures [11], which then makes it more difficult for them to pay for HI. Yet at the same time, without HI, they are much less equipped to be able to pay for such health expenditures. Since uninsured people incur considerable barriers to accessing substance abuse services [22], increasing access to HI for this population should continue to be a major priority among the Vietnamese government.

### Barriers and associated factors

Several barriers to and facilitators of accessing HI among MMT patients were identified in this study. Older patients were more likely to have sufficient information about HI than younger patients. This correlates with previous research, which has found that older methadone patients are more likely to have multiple comorbidies requiring additional care [23, 24].

Interestingly, we found that HIV-positive participants were more likely than HIV-negative participants to have sufficient information about HI, implying that they are more diligent about seeking out HI services. Previous studies have found that individuals who are HIV-positive are more likely to remain in MMT therapy [25]. Indeed, HIV-positive patients in Vietnam and other countries in Southeast Asia face a high financial burden [6, 26], which may then make them more likely to seek out information about how to access HI and get assistance in paying for their care.

In addition, we also found that unemployment was negatively associated with an individual’s level of insurance coverage. Several previous studies have found similar correlations between level of employment and rates of insurance coverage [27, 28]. Despite Vietnam’s push to increase universal HI for all citizens, there continues to be large swaths of the community that are not enrolled in HI, particularly among vulnerable populations like current and former injection drug users.

### Implications

Our study highlights the pressing need for increased HI enrollment among MMT patients in Vietnam, and also sheds light on possible solutions. There are a few ways to increase insurance enrollment among MMT patients in Vietnam. One way is to enroll patients in HI when they begin their methadone treatment. This strategy was employed by Umbricht-Schneiter, et al., in the work that they did with MMT patients in a research clinic in southeaster Baltimore, Maryland, USA [29]. Enrolling patients in HI as soon as they begin their MMT can help to ensure that patients remain engaged in their care and provides an easy access point to help answer any questions that patients have as they go through the sign-up process. MMT clinics across Vietnam should invest in social workers and/or other health staff to help patients sign up for HI. In addition, because our findings revealed patients from rural parts of Vietnam have more difficulty utilizing HI than individuals in urban parts of the country, rural MMT clinics should receive additional support staff to help patients there enroll in HI. Supporting and encouraging families who are affected by drug addiction to buy family health insurance plan for the whole family.

In addition to expanding government-based HI coverage options, another solution might be to increase employment opportunities for patients enrolled in MMT. That way, MMT patients may be able to receive HI from their employers, and will also have additional funds to pay for HI and other health-related expenditures that arise.

### Strengths and limitations

There are several strengths of this study, which should be noted. We were able to sample a wide swath of individuals enrolled in MMT in both rural and urban settings in northern Vietnam, who have never been surveyed previously about their utilization of and access to health insurance. We were also able to measure several aspects of both access to and ease of use of HI among this population and their families. Yet there were also a few limitations. A convenience sampling methods was employed, and as such our findings may not be representative of all MMT patients in Vietnam. In addition, we relied on patient recall of both their own experiences with drug use, MMT therapy, and HI, and the experiences of their family members. This might open the possibility of recall bias. However, despite these limitations, our study highlights important barriers to and facilitators of accessing HI among MMT patients in northern Vietnam.

## Conclusions

In summary, in this present study, we explored rates of HI and barriers to enrollment in HI among MMT patients in urban and rural parts of northern Vietnam. We found that the majority of patients were not enrolled in HI, largely due to financial constraints. Proposed solutions to increase enrollment include enrolling patients in HI when they begin MMT therapy and increasing employment opportunities for MMT patients. Because drug-addicted individuals face unique financial and health-related hardships, it is incredibly important to increase access to HI for these individuals.

## Abbreviation

EQ5D5L: EuroQol – 5 dimensions – 5 levels
HI: Health insurance
MMT: Methadone maintenance treatment
